# Schrödinger’s Worker: Are They Positive or Negative for SARS-CoV-2?

**DOI:** 10.3390/ijerph17176316

**Published:** 2020-08-31

**Authors:** Federico Meloni, Marcello Campagna, Angelo Restivo, Pierluigi Cocco

**Affiliations:** 1 Department of Medical Sciences and Public Health, Occupational Medicine Unit, University of Cagliari, SS 554, km 4.500, 09042 Monserrato (Cagliari), Italy; mcampagna@unica.it (M.C.); pcocco@unica.it (P.C.); 2 Department of Surgery, Colorectal Surgery Center, University of Cagliari, SS 554, km 4.500, 09042 Monserrato (Cagliari), Italy; arestivo@unica.it

**Keywords:** SARS-CoV-2, COVID-19, serologic tests, reverse transcriptase polymerase chain reaction, sensitivity and specificity, Predictive value of tests, occupational health, public health

## Abstract

In these days of 2020, tests for the diagnosis of SARS-CoV-2, and their use in the context of health surveillance of workers, are becoming popular. Nevertheless, their sensitivity and specificity could vary on the basis of the type of test used and on the moment of infection of the subject tested. The aim of this viewpoint paper is to make employers, workers, occupational physicians, and public health specialists think about the limits of diagnostic tests currently available, and the possible implication related to the erroneous and incautious assignment of “immunity passports” or “risk-free certificates” to workers during screening campaigns in workplaces.

Since SARS-CoV-2 made its appearance and began to spread worldwide causing hundreds of thousand deaths, many authors envisaged the occupational health and safety implications of the epidemic [1,2]. Many workers run a high risk of becoming infected, as well as being carriers themselves. This is especially true for some jobs, such as healthcare workers. In fact, being on the front line, they have a high risk of SARS-CoV-2 infection, of developing COVID-19, and of being a source of contagion for their patients, their colleagues, and their relatives [2]. Many possible factors contribute to COVID-19 clusters among healthcare workers: insufficient or incorrect use of protective personal equipment; close or direct contact with SARS-CoV-2 positive patients; working in confined indoor spaces; and shared canteen space, staff accommodation, transport, and/or social activities [3].

However, Healthcare is not the only sector where workers present a high risk of becoming infected: firefighters, policemen, cleaners, workers employed in care for the elderly, childcare, or education, public transport and taxi drivers, and many others run a significant risk [4]. Factors that increase risk of infection in the above mentioned and in many others work activities were described: working where interpersonal contact between workers is unavoidable; lack of compliance with preventive measures; sharing the same office space or canteen space, the same production line, dressing rooms, and accommodation (sometimes overfull and with poor hygiene conditions); meetings in overcrowd rooms; shared transport; working with clients; lack of facilities to wash hands; language challenges among migrant workers [3].

During the lockdown phase in some countries, only workers engaged in dealing with the COVID-19 emergency and those engaged in essential services were highly exposed; however, after the lockdown phase, millions of workers came back to work.

Employers, and occupational health services, therefore, face three major dilemmas. First: how reliably can we determine whether a worker is currently positive for SARS-CoV-2, and whether they can be a source of contagion for their own colleagues and for the people with whom they come in contact with while on duty? Second: how reliably can we establish whether a worker was previously infected and be assured that they will be indefinitely immune? Third, and most important: how can we reduce the risk of a worker getting the SARS-CoV-2 infection at work?

The aim of this viewpoint is to make the parties involved (employers, workers, occupational physicians, public health specialists) think about the limits of diagnostic tests currently available, and on the possible implication related to the erroneous, and incautious, assignment of “immunity passports” or “risk-free certificates” to workers during screening campaigns in workplaces.

To illustrate the quantum mechanics, the Nobel Prize physicist Erwin Schrödinger used a thought experiment, best known as the Schrödinger’s cat paradox. He invited his colleagues to image a cat inside a box. The box would also enclose a mechanism that would release a poison and kill the feline with a 50% probability. It is impossible to know if the cat is dead or alive before opening the box. The feline is in an indeterminate state, both dead and alive. If we do not make a measurement, multiple realities may exist at the same time.

Although the probability of SARS-CoV-2 infection is considerably lower than 50%, the worker is in an indeterminate situation as well, which probably would depend on the prevalence of infection among the general population, the pandemic’s curve at the time of observation, and the geographical area. For instance, two surveys conducted in Iceland in March on the general population revealed that, respectively, 0.8% (95% confidence interval (CI), 0.6–1.0%) and 0.6% (95% CI 0.3–0.9%) tested positive for SARS-CoV-2 infection [5]. In a pilot survey conducted in the United Kingdom, from 11th to 24th May, 0.24% (95% CI 0.11–0.46%) of the community population was estimated to be infected [6].

Therefore, to answer the first question “is it possible to determine with certainty whether a worker is currently positive or negative”, all we have to do is open the “box”. However, unlike the mentioned paradox, the judgment on whether “Schrödinger’s worker” is positive or negative would vary by time and space, and the safety probability threshold one would establish to make this choice.

In fact, the sensitivity and specificity of the currently available tests to measure specific IgM and IgG could vary substantially by type of serological test and week of infection [7], and they need further validation to verify their reliability and accuracy [8]. Moreover, a recent meta-analysis showed a pooled sensitivity of 64.8% (95% CI 54.5–74.0%) in rapid testing for SARS-CoV-2, based on the reverse-transcription polymerase chain reaction (RT-PCR) in respiratory samples [9]. This means that the false negative rate (the measure of workers that are infected but that results wrongly negative after being subjected to rapid test) would be, in absolute terms, unacceptably high to allow a safe admission to the workplace on the basis of one single rapid SARS-CoV-2 test. Indeed, giving a worker a “certificate of negativity” incorrectly, without further precautions, would expose their colleagues and/or the public to risk of contagion. Besides a low sensitivity [10], nasopharyngeal swabs often require some time for processing and issuing the results, depending on the number of tests being conducted. New, faster tests and different biological matrices (e.g., saliva) are currently being tested. However, sensitivity and specificity calculations in asymptomatic carriers are not yet available [11].

To open the “box”, through the use of the tests currently available in addition to collecting a detailed medical history (e.g., history of fever or respiratory symptoms in the previous weeks, new loss of taste or smell, cohabitation or contacts with subjects tested positive for SARS-CoV-2), might help to answer the second question: has the worker been infected in the past? However, again, the response we can get comes with a margin of error, due to different specificity by type of test used and little evidence about the time of seroconversion; also, evidence about protection from future infection and time of possible protective immunity is still missing [7,8,10]. Furthermore, recent studies suggested a rapid decay of anti–SARS-CoV-2 IgG levels in asymptomatic subjects [12] and in those with history of mild Covid-19 [13].

The third question, how can we deal with the challenge of protecting workers from SARS-CoV-2 infection during their work activity?

Primary prevention in the workplace could be the way forward: information and worker training about sources of exposure to SARS-CoV-2 and the hazards associated with exposure to the virus; use of appropriate workplace protocols to prevent or reduce the likelihood of exposure (social distancing, clean and dirty pathways, hand and workplace hygiene, practice of good respiratory etiquette, and procedures to isolate and identify suspected cases); use of adequate personal protective equipment, especially during procedures implying high risk (e.g., for health care workers); use of COVID-19 self-assessment tools; and identification of workers who may be at increased susceptibility [14,15,16]. In this regard, as the authors of a recent review and meta-analysis on this topic suggest, both FFP2 and surgical or 12–16-layer cotton masks are more protective than single-layer masks, especially in health care settings. They also suggest that physical distancing greater than 1 m and the use of eye protection may reduce the risk of infection. However, these interventions (even when appropriately used) seem not to be associated with complete protection; other measures, such as hand hygiene, are needed in addition to the use personal protective equipment [17].

These measures, many of which are also valid outside the workplace, could be accompanied by the above-mentioned tests even if, currently, they do not match the requirements for a good screening test that can “certificate negativity”. Moreover, there is no evidence for assignment of “immunity passports” or “risk-free certificates” in people who previously received a positive test [8,18]. These subjects, who incorrectly assume that they are immune to a second infection, might ignore public health advices and increase the risks of continued transmission in the workplace and at home [8].

Therefore, until a safe and effective vaccine becomes available, we will have “to open the box” and to shield as safely as reasonably possible its contents, if we want to preserve the health and wellbeing of workers and the community.
